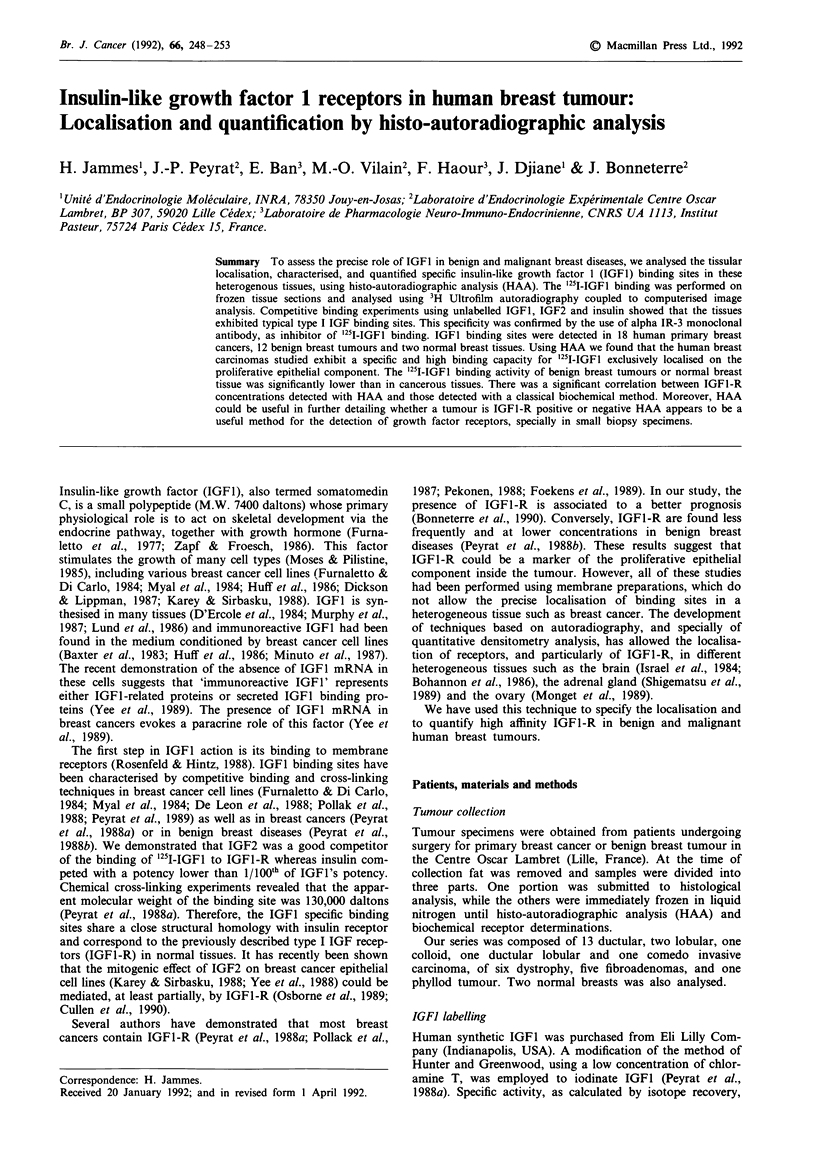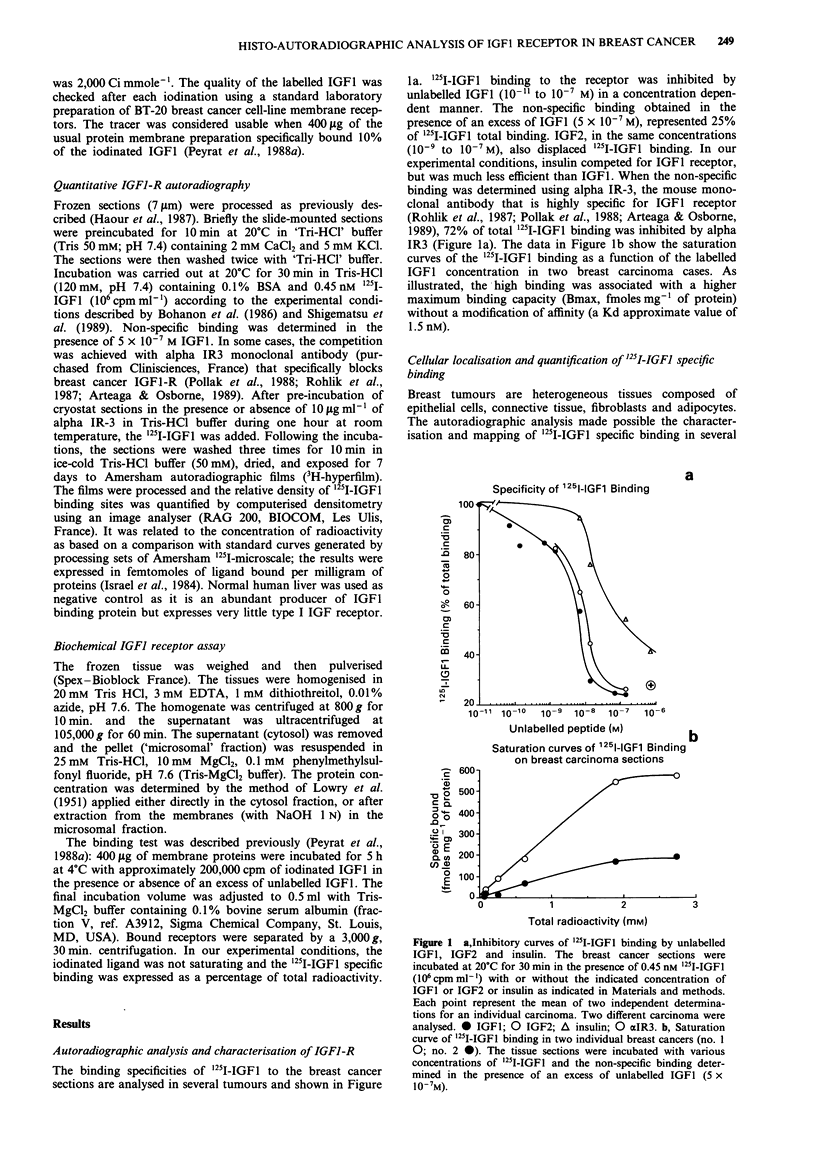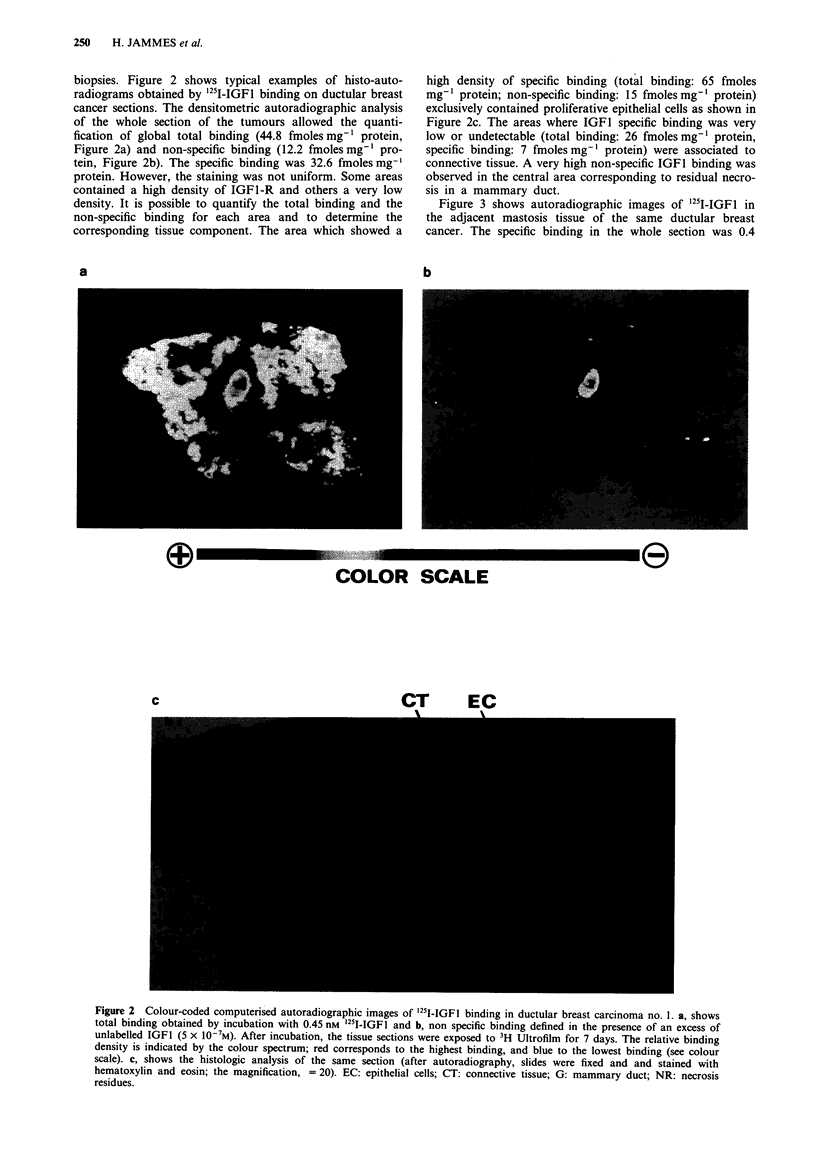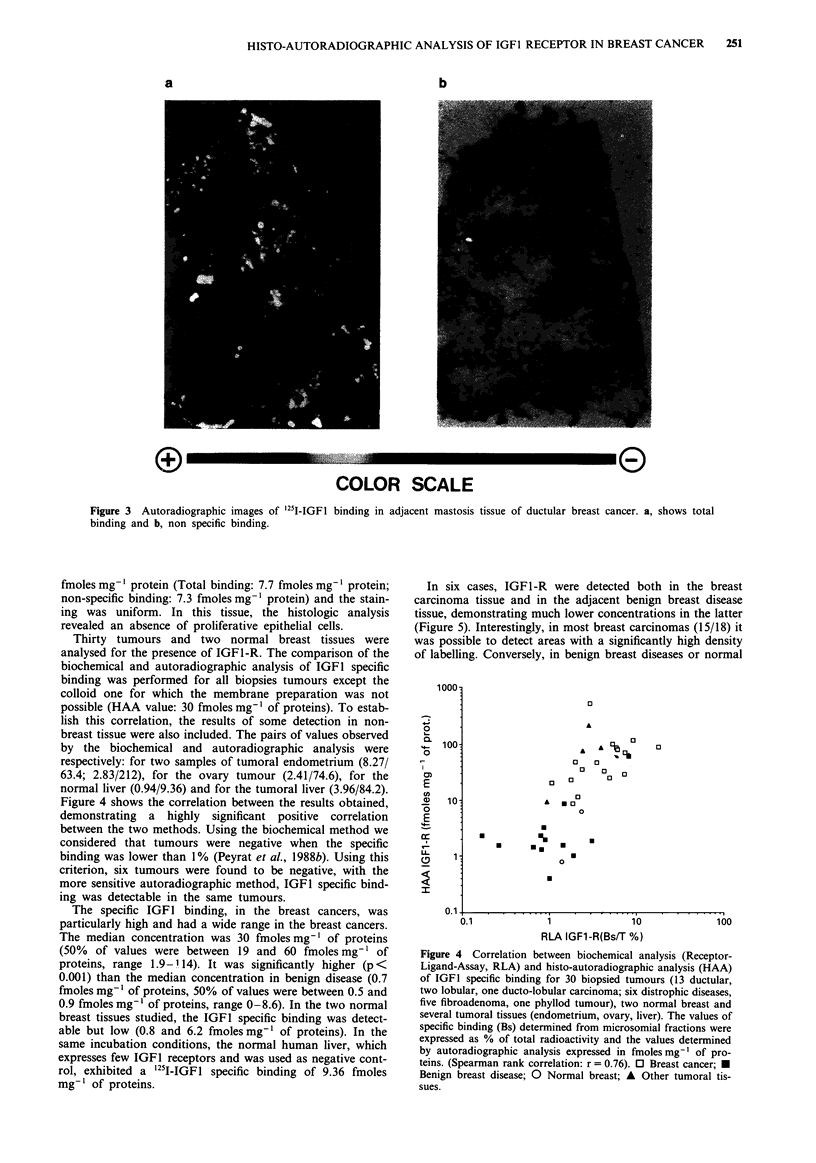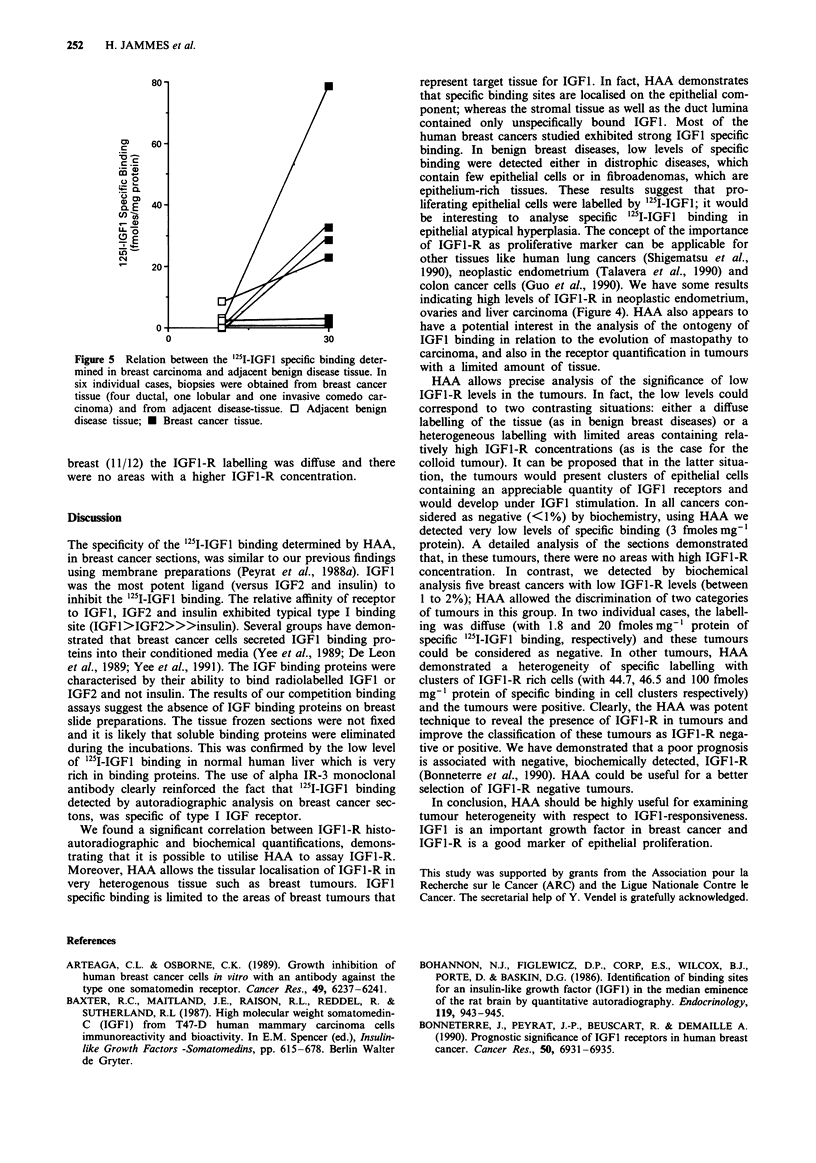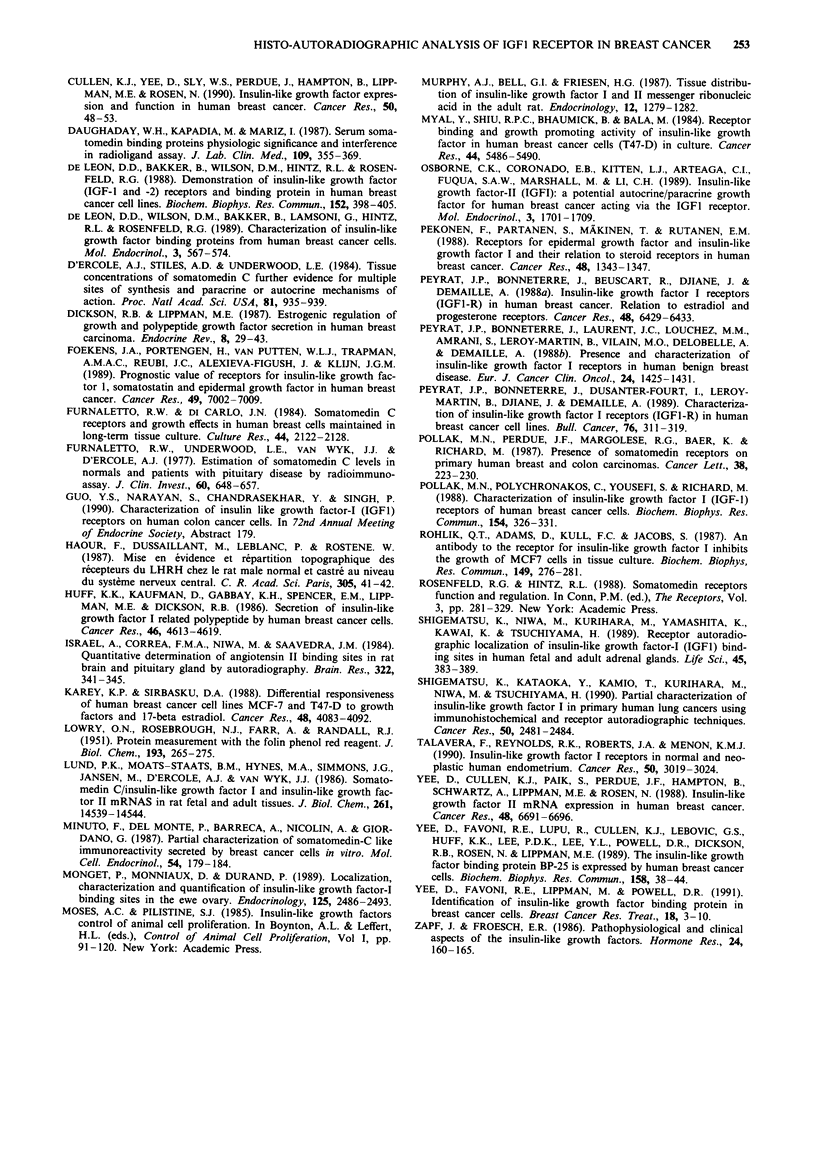# Insulin-like growth factor 1 receptors in human breast tumour: localisation and quantification by histo-autoradiographic analysis.

**DOI:** 10.1038/bjc.1992.252

**Published:** 1992-08

**Authors:** H. Jammes, J. P. Peyrat, E. Ban, M. O. Vilain, F. Haour, J. Djiane, J. Bonneterre

**Affiliations:** Unité d'Endocrinologie Moléculaire, INRA, Jouy-en-Josas, France.

## Abstract

**Images:**


Br. J. Cancer (1992), 66, 248-253 © Macmillan Press Ltd., 1992

### Insulin-like growth factor 1 receptors in human breast tumour: Localisation and quantification by histo-autoradiographic analysis

H. Jammes[1], J.-P. Peyrat[2], E. Ban[3], M.-O. Vilain[2], F. Haour[3], J. Djiane[1] & J. Bonneterre[2]

[1]*Unité d'Endocrinologie Moléculaire, INRA, 78350 Jouy-en-Josas;* [2]*Laboratoire d'Endocrinologie Expérimentale Centre Oscar Lambret, BP 307, 59020 Lille Cédex;* [3]*Laboratoire de Pharmacologie Neuro-Immuno-Endocrinienne, CNRS UA 1113, Institut Pasteur, 75724 Paris Cédex 15, France.*

**Summary** To assess the precise role of IGF1 in benign and malignant breast diseases, we analysed the tissular localisation, characterised, and quantified specific insulin-like growth factor 1 (IGF1) binding sites in these heterogenous tissues, using histo-autoradiographic analysis (HAA). The $^{125}$I-IGF1 binding was performed on frozen tissue sections and analysed using $^{3}$H Ultrofilm autoradiography coupled to computerised image analysis. Competitive binding experiments using unlabelled IGF1, IGF2 and insulin showed that the tissues exhibited typical type I IGF binding sites. This specificity was confirmed by the use of alpha IR-3 monoclonal antibody, as inhibitor of $^{125}$I-IGF1 binding. IGF1 binding sites were detected in 18 human primary breast cancers, 12 benign breast tumours and two normal breast tissues. Using HAA we found that the human breast carcinomas studied exhibit a specific and high binding capacity for $^{125}$I-IGF1 exclusively localised on the proliferative epithelial component. The $^{125}$I-IGF1 binding activity of benign breast tumours or normal breast tissue was significantly lower than in cancerous tissues. There was a significant correlation between IGF1-R concentrations detected with HAA and those detected with a classical biochemical method. Moreover, HAA could be useful in further detailing whether a tumour is IGF1-R positive or negative HAA appears to be a useful method for the detection of growth factor receptors, specially in small biopsy specimens.

Insulin-like growth factor (IGF1), also termed somatomedin C, is a small polypeptide (M.W. 7400 daltons) whose primary physiological role is to act on skeletal development via the endocrine pathway, together with growth hormone (Furnaletto *et al.*, 1977; Zapf & Froesch, 1986). This factor stimulates the growth of many cell types (Moses & Pilistine, 1985), including various breast cancer cell lines (Furnaletto & Di Carlo, 1984; Myal *et al.*, 1984; Huff *et al.*, 1986; Dickson & Lippman, 1987; Karey & Sirbasku, 1988). IGF1 is synthesised in many tissues (D'Ercole *et al.*, 1984; Murphy *et al.*, 1987; Lund *et al.*, 1986) and immunoreactive IGF1 had been found in the medium conditioned by breast cancer cell lines (Baxter *et al.*, 1983; Huff *et al.*, 1986; Minuto *et al.*, 1987). The recent demonstration of the absence of IGF1 mRNA in these cells suggests that 'immunoreactive IGF1' represents either IGF1-related proteins or secreted IGF1 binding proteins (Yee *et al.*, 1989). The presence of IGF1 mRNA in breast cancers evokes a paracrine role of this factor (Yee *et al.*, 1989).

The first step in IGF1 action is its binding to membrane receptors (Rosenfeld & Hintz, 1988). IGF1 binding sites have been characterised by competitive binding and cross-linking techniques in breast cancer cell lines (Furnaletto & Di Carlo, 1984; Myal *et al.*, 1984; De Leon *et al.*, 1988; Pollak *et al.*, 1988; Peyrat *et al.*, 1989) as well as in breast cancers (Peyrat *et al.*, 1988*a*) or in benign breast diseases (Peyrat *et al.*, 1988*b*). We demonstrated that IGF2 was a good competitor of the binding of $^{125}$I-IGF1 to IGF1-R whereas insulin competed with a potency lower than 1/100$^{th}$ of IGF1's potency. Chemical cross-linking experiments revealed that the apparent molecular weight of the binding site was 130,000 daltons (Peyrat *et al.*, 1988*a*). Therefore, the IGF1 specific binding sites share a close structural homology with insulin receptor and correspond to the previously described type I IGF receptors (IGF1-R) in normal tissues. It has recently been shown that the mitogenic effect of IGF2 on breast cancer epithelial cell lines (Karey & Sirbasku, 1988; Yee *et al.*, 1988) could be mediated, at least partially, by IGF1-R (Osborne *et al.*, 1989; Cullen *et al.*, 1990).

Several authors have demonstrated that most breast cancers contain IGF1-R (Peyrat *et al.*, 1988*a*; Pollack *et al.*, 1987; Pekonen, 1988; Foekens *et al.*, 1989). In our study, the presence of IGF1-R is associated to a better prognosis (Bonneterre *et al.*, 1990). Conversely, IGF1-R are found less frequently and at lower concentrations in benign breast diseases (Peyrat *et al.*, 1988*b*). These results suggest that IGF1-R could be a marker of the proliferative epithelial component inside the tumour. However, all of these studies had been performed using membrane preparations, which do not allow the precise localisation of binding sites in a heterogeneous tissue such as breast cancer. The development of techniques based on autoradiography, and specially of quantitative densitometry analysis, has allowed the localisation of receptors, and particularly of IGF1-R, in different heterogeneous tissues such as the brain (Israel *et al.*, 1984; Bohannon *et al.*, 1986), the adrenal gland (Shigematsu *et al.*, 1989) and the ovary (Monget *et al.*, 1989).

We have used this technique to specify the localisation and to quantify high affinity IGF1-R in benign and malignant human breast tumours.

### Patients, materials and methods

### *Tumour collection*

Tumour specimens were obtained from patients undergoing surgery for primary breast cancer or benign breast tumour in the Centre Oscar Lambret (Lille, France). At the time of collection fat was removed and samples were divided into three parts. One portion was submitted to histological analysis, while the others were immediately frozen in liquid nitrogen until histo-autoradiographic analysis (HAA) and biochemical receptor determinations.

Our series was composed of 13 ductular, two lobular, one colloid, one ductular lobular and one comedo invasive carcinoma, of six dystrophy, five fibroadenomas, and one phyllod tumour. Two normal breasts was also analysed.

### *IGF1 labelling*

Human synthetic IGF1 was purchased from Eli Lilly Company (Indianapolis, USA). A modification of the method of Hunter and Greenwood, using a low concentration of chloramine T, was employed to iodinate IGF1 (Peyrat *et al.*, 1988*a*). Specific activity, as calculated by isotope recovery,

Correspondence: H. Jammes.
Received 20 January 1992; and in revised form 1 April 1992.

was 2,000 Ci mmole$^{-1}$. The quality of the labelled IGF1 was checked after each iodination using a standard laboratory preparation of BT-20 breast cancer cell-line membrane receptors. The tracer was considered usable when 400 µg of the usual protein membrane preparation specifically bound 10% of the iodinated IGF1 (Peyrat *et al.*, 1988*a*).

*Quantitative IGF1-R autoradiography*

Frozen sections (7 µm) were processed as previously described (Haour *et al.*, 1987). Briefly the slide-mounted sections were preincubated for 10 min at 20°C in 'Tri-HCl' buffer (Tris 50 mM; pH 7.4) containing 2 mM $CaCl_2$ and 5 mM KCl. The sections were then washed twice with 'Tri-HCl' buffer. Incubation was carried out at 20°C for 30 min in Tris-HCl (120 mM, pH 7.4) containing 0.1% BSA and 0.45 nM $^{125}$I-IGF1 ($10^6$ cpm ml$^{-1}$) according to the experimental conditions described by Bohanon *et al.* (1986) and Shigematsu *et al.* (1989). Non-specific binding was determined in the presence of $5 \times 10^{-7}$ M IGF1. In some cases, the competition was achieved with alpha IR3 monoclonal antibody (purchased from Clinisciences, France) that specifically blocks breast cancer IGF1-R (Pollak *et al.*, 1988; Rohlik *et al.*, 1987; Arteaga & Osborne, 1989). After pre-incubation of cryostat sections in the presence or absence of 10 µg ml$^{-1}$ of alpha IR-3 in Tris-HCl buffer during one hour at room temperature, the $^{125}$I-IGF1 was added. Following the incubations, the sections were washed three times for 10 min in ice-cold Tris-HCl buffer (50 mM), dried, and exposed for 7 days to Amersham autoradiographic films ($^3$H-hyperfilm). The films were processed and the relative density of $^{125}$I-IGF1 binding sites was quantified by computerised densitometry using an image analyser (RAG 200, BIOCOM, Les Ulis, France). It was related to the concentration of radioactivity as based on a comparison with standard curves generated by processing sets of Amersham $^{125}$I-microscale; the results were expressed in femtomoles of ligand bound per milligram of proteins (Israel *et al.*, 1984). Normal human liver was used as negative control as it is an abundant producer of IGF1 binding protein but expresses very little type I IGF receptor.

*Biochemical IGF1 receptor assay*

The frozen tissue was weighed and then pulverised (Spex–Bioblock France). The tissues were homogenised in 20 mM Tris HCl, 3 mM EDTA, 1 mM dithiothreitol, 0.01% azide, pH 7.6. The homogenate was centrifuged at 800 *g* for 10 min. and the supernatant was ultracentrifuged at 105,000 *g* for 60 min. The supernatant (cytosol) was removed and the pellet ('microsomal' fraction) was resuspended in 25 mM Tris-HCl, 10 mM $MgCl_2$, 0.1 mM phenylmethylsulfonyl fluoride, pH 7.6 (Tris-$MgCl_2$ buffer). The protein concentration was determined by the method of Lowry *et al.* (1951) applied either directly in the cytosol fraction, or after extraction from the membranes (with NaOH 1 N) in the microsomal fraction.

The binding test was described previously (Peyrat *et al.*, 1988*a*): 400 µg of membrane proteins were incubated for 5 h at 4°C with approximately 200,000 cpm of iodinated IGF1 in the presence or absence of an excess of unlabelled IGF1. The final incubation volume was adjusted to 0.5 ml with Tris-$MgCl_2$ buffer containing 0.1% bovine serum albumin (fraction V, ref. A3912, Sigma Chemical Company, St. Louis, MD, USA). Bound receptors were separated by a 3,000 *g*, 30 min. centrifugation. In our experimental conditions, the iodinated ligand was not saturating and the $^{125}$I-IGF1 specific binding was expressed as a percentage of total radioactivity.

### Results

*Autoradiographic analysis and characterisation of IGF1-R*

The binding specificities of $^{125}$I-IGF1 to the breast cancer sections are analysed in several tumours and shown in Figure 1a. $^{125}$I-IGF1 binding to the receptor was inhibited by unlabelled IGF1 ($10^{-11}$ to $10^{-7}$ M) in a concentration dependent manner. The non-specific binding obtained in the presence of an excess of IGF1 ($5 \times 10^{-7}$ M), represented 25% of $^{125}$I-IGF1 total binding. IGF2, in the same concentrations ($10^{-9}$ to $10^{-7}$ M), also displaced $^{125}$I-IGF1 binding. In our experimental conditions, insulin competed for IGF1 receptor, but was much less efficient than IGF1. When the non-specific binding was determined using alpha IR-3, the mouse monoclonal antibody that is highly specific for IGF1 receptor (Rohlik *et al.*, 1987; Pollak *et al.*, 1988; Arteaga & Osborne, 1989), 72% of total $^{125}$I-IGF1 binding was inhibited by alpha IR3 (Figure 1a). The data in Figure 1b show the saturation curves of the $^{125}$I-IGF1 binding as a function of the labelled IGF1 concentration in two breast carcinoma cases. As illustrated, the high binding was associated with a higher maximum binding capacity (Bmax, fmoles mg$^{-1}$ of protein) without a modification of affinity (a Kd approximate value of 1.5 nM).

*Cellular localisation and quantification of $^{125}$I-IGF1 specific binding*

Breast tumours are heterogeneous tissues composed of epithelial cells, connective tissue, fibroblasts and adipocytes. The autoradiographic analysis made possible the characterisation and mapping of $^{125}$I-IGF1 specific binding in several

**Figure 1** **a**, Inhibitory curves of $^{125}$I-IGF1 binding by unlabelled IGF1, IGF2 and insulin. The breast cancer sections were incubated at 20°C for 30 min in the presence of 0.45 nM $^{125}$I-IGF1 ($10^6$ cpm ml$^{-1}$) with or without the indicated concentration of IGF1 or IGF2 or insulin as indicated in Materials and methods. Each point represent the mean of two independent determinations for an individual carcinoma. Two different carcinoma were analysed. ● IGF1; ○ IGF2; Δ insulin; ○ αIR3. **b**, Saturation curve of $^{125}$I-IGF1 binding in two individual breast cancers (no. 1 ○; no. 2 ●). The tissue sections were incubated with various concentrations of $^{125}$I-IGF1 and the non-specific binding determined in the presence of an excess of unlabelled IGF1 ($5 \times 10^{-7}$M).

biopsies. Figure 2 shows typical examples of histo-autoradiograms obtained by $^{125}$I-IGF1 binding on ductular breast cancer sections. The densitometric autoradiographic analysis of the whole section of the tumours allowed the quantification of global total binding (44.8 fmoles mg$^{-1}$ protein, Figure 2a) and non-specific binding (12.2 fmoles mg$^{-1}$ protein, Figure 2b). The specific binding was 32.6 fmoles mg$^{-1}$ protein. However, the staining was not uniform. Some areas contained a high density of IGF1-R and others a very low density. It is possible to quantify the total binding and the non-specific binding for each area and to determine the corresponding tissue component. The area which showed a high density of specific binding (total binding: 65 fmoles mg$^{-1}$ protein; non-specific binding: 15 fmoles mg$^{-1}$ protein) exclusively contained proliferative epithelial cells as shown in Figure 2c. The areas where IGF1 specific binding was very low or undetectable (total binding: 26 fmoles mg$^{-1}$ protein, specific binding: 7 fmoles mg$^{-1}$ protein) were associated to connective tissue. A very high non-specific IGF1 binding was observed in the central area corresponding to residual necrosis in a mammary duct.

Figure 3 shows autoradiographic images of $^{125}$I-IGF1 in the adjacent mastosis tissue of the same ductular breast cancer. The specific binding in the whole section was 0.4

**Figure 2** Colour-coded computerised autoradiographic images of $^{125}$I-IGF1 binding in ductular breast carcinoma no. 1. **a**, shows total binding obtained by incubation with 0.45 nM $^{125}$I-IGF1 and **b**, non specific binding defined in the presence of an excess of unlabelled IGF1 ($5 \times 10^{-7}$M). After incubation, the tissue sections were exposed to $^{3}$H Ultrofilm for 7 days. The relative binding density is indicated by the colour spectrum; red corresponds to the highest binding, and blue to the lowest binding (see colour scale). **c**, shows the histologic analysis of the same section (after autoradiography, slides were fixed and and stained with hematoxylin and eosin; the magnification, = 20). EC: epithelial cells; CT: connective tissue; G: mammary duct; NR: necrosis residues.

**Figure 3** Autoradiographic images of $^{125}$I-IGF1 binding in adjacent mastosis tissue of ductular breast cancer. **a**, shows total binding and **b**, non specific binding.

fmoles mg$^{-1}$ protein (Total binding: 7.7 fmoles mg$^{-1}$ protein; non-specific binding: 7.3 fmoles mg$^{-1}$ protein) and the staining was uniform. In this tissue, the histologic analysis revealed an absence of proliferative epithelial cells.

Thirty tumours and two normal breast tissues were analysed for the presence of IGF1-R. The comparison of the biochemical and autoradiographic analysis of IGF1 specific binding was performed for all biopsies tumours except the colloid one for which the membrane preparation was not possible (HAA value: 30 fmoles mg$^{-1}$ of proteins). To establish this correlation, the results of some detection in non-breast tissue were also included. The pairs of values observed by the biochemical and autoradiographic analysis were respectively: for two samples of tumoral endometrium (8.27/63.4; 2.83/212), for the ovary tumour (2.41/74.6), for the normal liver (0.94/9.36) and for the tumoral liver (3.96/84.2). Figure 4 shows the correlation between the results obtained, demonstrating a highly significant positive correlation between the two methods. Using the biochemical method we considered that tumours were negative when the specific binding was lower than 1% (Peyrat *et al.*, 1988*b*). Using this criterion, six tumours were found to be negative, with the more sensitive autoradiographic method, IGF1 specific binding was detectable in the same tumours.

The specific IGF1 binding, in the breast cancers, was particularly high and had a wide range in the breast cancers. The median concentration was 30 fmoles mg$^{-1}$ of proteins (50% of values were between 19 and 60 fmoles mg$^{-1}$ of proteins, range 1.9–114). It was significantly higher ($p < 0.001$) than the median concentration in benign disease (0.7 fmoles mg$^{-1}$ of proteins, 50% of values were between 0.5 and 0.9 fmoles mg$^{-1}$ of proteins, range 0–8.6). In the two normal breast tissues studied, the IGF1 specific binding was detectable but low (0.8 and 6.2 fmoles mg$^{-1}$ of proteins). In the same incubation conditions, the normal human liver, which expresses few IGF1 receptors and was used as negative control, exhibited a $^{125}$I-IGF1 specific binding of 9.36 fmoles mg$^{-1}$ of proteins.

In six cases, IGF1-R were detected both in the breast carcinoma tissue and in the adjacent benign breast disease tissue, demonstrating much lower concentrations in the latter (Figure 5). Interestingly, in most breast carcinomas (15/18) it was possible to detect areas with a significantly high density of labelling. Conversely, in benign breast diseases or normal

**Figure 4** Correlation between biochemical analysis (Receptor-Ligand-Assay, RLA) and histo-autoradiographic analysis (HAA) of IGF1 specific binding for 30 biopsied tumours (13 ductular, two lobular, one ducto-lobular carcinoma; six distrophic diseases, five fibroadenoma, one phyllod tumour), two normal breast and several tumoral tissues (endometrium, ovary, liver). The values of specific binding (Bs) determined from microsomial fractions were expressed as % of total radioactivity and the values determined by autoradiographic analysis expressed in fmoles mg$^{-1}$ of proteins. (Spearman rank correlation: r = 0.76). □ Breast cancer; ■ Benign breast disease; ○ Normal breast; ▲ Other tumoral tissues.

**Figure 5** Relation between the $^{125}$I-IGF1 specific binding determined in breast carcinoma and adjacent benign disease tissue. In six individual cases, biopsies were obtained from breast cancer tissue (four ductal, one lobular and one invasive comedo carcinoma) and from adjacent disease-tissue. □ Adjacent benign disease tissue; ■ Breast cancer tissue.

breast (11/12) the IGF1-R labelling was diffuse and there were no areas with a higher IGF1-R concentration.

### Discussion

The specificity of the $^{125}$I-IGF1 binding determined by HAA, in breast cancer sections, was similar to our previous findings using membrane preparations (Peyrat *et al.*, 1988*a*). IGF1 was the most potent ligand (versus IGF2 and insulin) to inhibit the $^{125}$I-IGF1 binding. The relative affinity of receptor to IGF1, IGF2 and insulin exhibited typical type I binding site (IGF1>IGF2>>>insulin). Several groups have demonstrated that breast cancer cells secreted IGF1 binding proteins into their conditioned media (Yee *et al.*, 1989; De Leon *et al.*, 1989; Yee *et al.*, 1991). The IGF binding proteins were characterised by their ability to bind radiolabelled IGF1 or IGF2 and not insulin. The results of our competition binding assays suggest the absence of IGF binding proteins on breast slide preparations. The tissue frozen sections were not fixed and it is likely that soluble binding proteins were eliminated during the incubations. This was confirmed by the low level of $^{125}$I-IGF1 binding in normal human liver which is very rich in binding proteins. The use of alpha IR-3 monoclonal antibody clearly reinforced the fact that $^{125}$I-IGF1 binding detected by autoradiographic analysis on breast cancer sectons, was specific of type I IGF receptor.

We found a significant correlation between IGF1-R histoautoradiographic and biochemical quantifications, demonstrating that it is possible to utilise HAA to assay IGF1-R. Moreover, HAA allows the tissular localisation of IGF1-R in very heterogenous tissue such as breast tumours. IGF1 specific binding is limited to the areas of breast tumours that represent target tissue for IGF1. In fact, HAA demonstrates that specific binding sites are localised on the epithelial component; whereas the stromal tissue as well as the duct lumina contained only unspecifically bound IGF1. Most of the human breast cancers studied exhibited strong IGF1 specific binding. In benign breast diseases, low levels of specific binding were detected either in distrophic diseases, which contain few epithelial cells or in fibroadenomas, which are epithelium-rich tissues. These results suggest that proliferating epithelial cells were labelled by $^{125}$I-IGF1; it would be interesting to analyse specific $^{125}$I-IGF1 binding in epithelial atypical hyperplasia. The concept of the importance of IGF1-R as proliferative marker can be applicable for other tissues like human lung cancers (Shigematsu *et al.*, 1990), neoplastic endometrium (Talavera *et al.*, 1990) and colon cancer cells (Guo *et al.*, 1990). We have some results indicating high levels of IGF1-R in neoplastic endometrium, ovaries and liver carcinoma (Figure 4). HAA also appears to have a potential interest in the analysis of the ontogeny of IGF1 binding in relation to the evolution of mastopathy to carcinoma, and also in the receptor quantification in tumours with a limited amount of tissue.

HAA allows precise analysis of the significance of low IGF1-R levels in the tumours. In fact, the low levels could correspond to two contrasting situations: either a diffuse labelling of the tissue (as in benign breast diseases) or a heterogeneous labelling with limited areas containing relatively high IGF1-R concentrations (as is the case for the colloid tumour). It can be proposed that in the latter situation, the tumours would present clusters of epithelial cells containing an appreciable quantity of IGF1 receptors and would develop under IGF1 stimulation. In all cancers considered as negative (<1%) by biochemistry, using HAA we detected very low levels of specific binding (3 fmoles mg$^{-1}$ protein). A detailed analysis of the sections demonstrated that, in these tumours, there were no areas with high IGF1-R concentration. In contrast, we detected by biochemical analysis five breast cancers with low IGF1-R levels (between 1 to 2%); HAA allowed the discrimination of two categories of tumours in this group. In two individual cases, the labelling was diffuse (with 1.8 and 20 fmoles mg$^{-1}$ protein of specific $^{125}$I-IGF1 binding, respectively) and these tumours could be considered as negative. In other tumours, HAA demonstrated a heterogeneity of specific labelling with clusters of IGF1-R rich cells (with 44.7, 46.5 and 100 fmoles mg$^{-1}$ protein of specific binding in cell clusters respectively) and the tumours were positive. Clearly, the HAA was potent technique to reveal the presence of IGF1-R in tumours and improve the classification of these tumours as IGF1-R negative or positive. We have demonstrated that a poor prognosis is associated with negative, biochemically detected, IGF1-R (Bonneterre *et al.*, 1990). HAA could be useful for a better selection of IGF1-R negative tumours.

In conclusion, HAA should be highly useful for examining tumour heterogeneity with respect to IGF1-responsiveness. IGF1 is an important growth factor in breast cancer and IGF1-R is a good marker of epithelial proliferation.

This study was supported by grants from the Association pour la Recherche sur le Cancer (ARC) and the Ligue Nationale Contre le Cancer. The secretarial help of Y. Vendel is gratefully acknowledged.

### References

ARTEAGA, C.L. & OSBORNE, C.K. (1989). Growth inhibition of human breast cancer cells *in vitro* with an antibody against the type one somatomedin receptor. *Cancer Res.*, **49**, 6237-6241.

BAXTER, R.C., MAITLAND, J.E., RAISON, R.L., REDDEL, R. & SUTHERLAND, R.L (1987). High molecular weight somatomedin-C (IGF1) from T47-D human mammary carcinoma cells immunoreactivity and bioactivity. In E.M. Spencer (ed.), *Insulin-like Growth Factors -Somatomedins*, pp. 615-678. Berlin Walter de Gryter.

BOHANNON, N.J., FIGLEWICZ, D.P., CORP, E.S., WILCOX, B.J., PORTE, D. & BASKIN, D.G. (1986). Identification of binding sites for an insulin-like growth factor (IGF1) in the median eminence of the rat brain by quantitative autoradiography. *Endocrinology*, **119**, 943-945.

BONNETERRE, J., PEYRAT, J.-P., BEUSCART, R. & DEMAILLE A. (1990). Prognostic significance of IGF1 receptors in human breast cancer. *Cancer Res.*, **50**, 6931-6935.

CULLEN, K.J., YEE, D., SLY, W.S., PERDUE, J., HAMPTON, B., LIPPMAN, M.E. & ROSEN, N. (1990). Insulin-like growth factor expression and function in human breast cancer. *Cancer Res.*, **50**, 48–53.

DAUGHADAY, W.H., KAPADIA, M. & MARIZ, I. (1987). Serum somatomedin binding proteins physiologic significance and interference in radioligand assay. *J. Lab. Clin. Med.*, **109**, 355–369.

DE LEON, D.D., BAKKER, B., WILSON, D.M., HINTZ, R.L. & ROSENFELD, R.G. (1988). Demonstration of insulin-like growth factor (IGF-1 and -2) receptors and binding protein in human breast cancer cell lines. *Biochem. Biophys. Res. Commun.*, **152**, 398–405.

DE LEON, D.D., WILSON, D.M., BAKKER, B., LAMSONI, G., HINTZ, R.L. & ROSENFELD, R.G. (1989). Characterization of insulin-like growth factor binding proteins from human breast cancer cells. *Mol. Endocrinol.*, **3**, 567–574.

D'ERCOLE, A.J., STILES, A.D. & UNDERWOOD, L.E. (1984). Tissue concentrations of somatomedin C further evidence for multiple sites of synthesis and paracrine or autocrine mechanisms of action. *Proc. Natl Acad. Sci. USA*, **81**, 935–939.

DICKSON, R.B. & LIPPMAN, M.E. (1987). Estrogenic regulation of growth and polypeptide growth factor secretion in human breast carcinoma. *Endocrine Rev.*, **8**, 29–43.

FOEKENS, J.A., PORTENGEN, H., VAN PUTTEN, W.L.J., TRAPMAN, A.M.A.C., REUBI, J.C., ALEXIEVA-FIGUSH, J. & KLIJN, J.G.M. (1989). Prognostic value of receptors for insulin-like growth factor 1, somatostatin and epidermal growth factor in human breast cancer. *Cancer Res.*, **49**, 7002–7009.

FURNALETTO, R.W. & DI CARLO, J.N. (1984). Somatomedin C receptors and growth effects in human breast cells maintained in long-term tissue culture. *Culture Res.*, **44**, 2122–2128.

FURNALETTO, R.W., UNDERWOOD, L.E., VAN WYK, J.J. & D'ERCOLE, A.J. (1977). Estimation of somatomedin C levels in normals and patients with pituitary disease by radioimmunoassay. *J. Clin. Invest.*, **60**, 648–657.

GUO, Y.S., NARAYAN, S., CHANDRASEKHAR, Y. & SINGH, P. (1990). Characterization of insulin like growth factor-I (IGF1) receptors on human colon cancer cells. In *72nd Annual Meeting of Endocrine Society*, Abstract 179.

HAOUR, F., DUSSAILLANT, M., LEBLANC, P. & ROSTENE. W. (1987). Mise en évidence et répartition topographique des récepteurs du LHRH chez le rat male normal et castré au niveau du système nerveux central. *C. R. Acad. Sci. Paris*, **305**, 41–42.

HUFF, K.K., KAUFMAN, D., GABBAY, K.H., SPENCER, E.M., LIPPMAN, M.E. & DICKSON, R.B. (1986). Secretion of insulin-like growth factor I related polypeptide by human breast cancer cells. *Cancer Res.*, **46**, 4613–4619.

ISRAEL, A., CORREA, F.M.A., NIWA, M. & SAAVEDRA, J.M. (1984). Quantitative determination of angiotensin II binding sites in rat brain and pituitary gland by autoradiography. *Brain. Res.*, **322**, 341–345.

KAREY, K.P. & SIRBASKU, D.A. (1988). Differential responsiveness of human breast cancer cell lines MCF-7 and T47-D to growth factors and 17-beta estradiol. *Cancer Res.*, **48**, 4083–4092.

LOWRY, O.N., ROSEBROUGH, N.J., FARR, A. & RANDALL, R.J. (1951). Protein measurement with the folin phenol red reagent. *J. Biol. Chem.*, **193**, 265–275.

LUND, P.K., MOATS-STAATS, B.M., HYNES, M.A., SIMMONS, J.G., JANSEN, M., D'ERCOLE, A.J. & VAN WYK, J.J. (1986). Somatomedin C/insulin-like growth factor I and insulin-like growth factor II mRNAS in rat fetal and adult tissues. *J. Biol. Chem.*, **261**, 14539–14544.

MINUTO, F., DEL MONTE, P., BARRECA, A., NICOLIN, A. & GIORDANO, G. (1987). Partial characterization of somatomedin-C like immunoreactivity secreted by breast cancer cells *in vitro*. *Mol. Cell. Endocrinol.*, **54**, 179–184.

MONGET, P., MONNIAUX, D. & DURAND, P. (1989). Localization, characterization and quantification of insulin-like growth factor-I binding sites in the ewe ovary. *Endocrinology*, **125**, 2486–2493.

MOSES, A.C. & PILISTINE, S.J. (1985). Insulin-like growth factors control of animal cell proliferation. In Boynton, A.L. & Leffert, H.L. (eds.), *Control of Animal Cell Proliferation*, Vol I, pp. 91–120. New York: Academic Press.

MURPHY, A.J., BELL, G.I. & FRIESEN, H.G. (1987). Tissue distribution of insulin-like growth factor I and II messenger ribonucleic acid in the adult rat. *Endocrinology*, **12**, 1279–1282.

MYAL, Y., SHIU, R.P.C., BHAUMICK, B. & BALA, M. (1984). Receptor binding and growth promoting activity of insulin-like growth factor in human breast cancer cells (T47-D) in culture. *Cancer Res.*, **44**, 5486–5490.

OSBORNE, C.K., CORONADO, E.B., KITTEN, L.J., ARTEAGA, C.I., FUQUA, S.A.W., MARSHALL, M. & LI, C.H. (1989). Insulin-like growth factor-II (IGFI): a potential autocrine/paracrine growth factor for human breast cancer acting via the IGF1 receptor. *Mol. Endocrinol.*, **3**, 1701–1709.

PEKONEN, F., PARTANEN, S., MÄKINEN, T. & RUTANEN, E.M. (1988). Receptors for epidermal growth factor and insulin-like growth factor I and their relation to steroid receptors in human breast cancer. *Cancer Res.*, **48**, 1343–1347.

PEYRAT, J.P., BONNETERRE, J., BEUSCART, R., DJIANE, J. & DEMAILLE, A. (1988*a*). Insulin-like growth factor I receptors (IGF1-R) in human breast cancer. Relation to estradiol and progesterone receptors. *Cancer Res.*, **48**, 6429–6433.

PEYRAT, J.P., BONNETERRE, J., LAURENT, J.C., LOUCHEZ, M.M., AMRANI, S., LEROY-MARTIN, B., VILAIN, M.O., DELOBELLE, A. & DEMAILLE, A. (1988*b*). Presence and characterization of insulin-like growth factor I receptors in human benign breast disease. *Eur. J. Cancer Clin. Oncol.*, **24**, 1425–1431.

PEYRAT, J.P., BONNETERRE, J., DUSANTER-FOURT, I., LEROY-MARTIN, B., DJIANE, J. & DEMAILLE, A. (1989). Characterization of insulin-like growth factor I receptors (IGF1-R) in human breast cancer cell lines. *Bull. Cancer*, **76**, 311–319.

POLLAK, M.N., PERDUE, J.F., MARGOLESE, R.G., BAER, K. & RICHARD, M. (1987). Presence of somatomedin receptors on primary human breast and colon carcinomas. *Cancer Lett.*, **38**, 223–230.

POLLAK, M.N., POLYCHRONAKOS, C., YOUSEFI, S. & RICHARD, M. (1988). Characterization of insulin-like growth factor I (IGF-1) receptors of human breast cancer cells. *Biochem. Biophys. Res. Commun.*, **154**, 326–331.

ROHLIK, Q.T., ADAMS, D., KULL, F.C. & JACOBS, S. (1987). An antibody to the receptor for insulin-like growth factor I inhibits the growth of MCF7 cells in tissue culture. *Biochem. Biophys, Res. Commun.*, **149**, 276–281.

ROSENFELD, R.G. & HINTZ, R.L. (1988). Somatomedin receptors function and regulation. In Conn, P.M. (ed.), *The Receptors*, Vol. 3, pp. 281–329. New York: Academic Press.

SHIGEMATSU, K., NIWA, M., KURIHARA, M., YAMASHITA, K., KAWAI, K. & TSUCHIYAMA, H. (1989). Receptor autoradiographic localization of insulin-like growth factor-I (IGF1) binding sites in human fetal and adult adrenal glands. *Life Sci.*, **45**, 383–389.

SHIGEMATSU, K., KATAOKA, Y., KAMIO, T., KURIHARA, M., NIWA, M. & TSUCHIYAMA, H. (1990). Partial characterization of insulin-like growth factor I in primary human lung cancers using immunohistochemical and receptor autoradiographic techniques. *Cancer Res.*, **50**, 2481–2484.

TALAVERA, F., REYNOLDS, R.K., ROBERTS, J.A. & MENON, K.M.J. (1990). Insulin-like growth factor I receptors in normal and neoplastic human endometrium. *Cancer Res.*, **50**, 3019–3024.

YEE, D., CULLEN, K.J., PAIK, S., PERDUE, J.F., HAMPTON, B., SCHWARTZ, A., LIPPMAN, M.E. & ROSEN, N. (1988). Insulin-like growth factor II mRNA expression in human breast cancer. *Cancer Res.*, **48**, 6691–6696.

YEE, D., FAVONI, R.E., LUPU, R., CULLEN, K.J., LEBOVIC, G.S., HUFF, K.K., LEE, P.D.K., LEE, Y.L., POWELL, D.R., DICKSON, R.B., ROSEN, N. & LIPPMAN, M.E. (1989). The insulin-like growth factor binding protein BP-25 is expressed by human breast cancer cells. *Biochem. Biophys. Res. Commun.*, **158**, 38–44.

YEE, D., FAVONI, R.E., LIPPMAN, M. & POWELL, D.R. (1991). Identification of insulin-like growth factor binding protein in breast cancer cells. *Breast Cancer Res. Treat.*, **18**, 3–10.

ZAPF, J. & FROESCH, E.R. (1986). Pathophysiological and clinical aspects of the insulin-like growth factors. *Hormone Res.*, **24**, 160–165.